# Development and validation of a prognostic index to predict pulmonary metastasis of giant cell tumor of bone

**DOI:** 10.18632/oncotarget.22478

**Published:** 2017-11-08

**Authors:** Bo Wang, Wei Chen, Xianbiao Xie, Jian Tu, Gang Huang, Changye Zou, Junqiang Yin, Lili Wen, Jingnan Shen

**Affiliations:** ^1^ Musculoskeletal Oncology Center, The First Affiliated Hospital of Sun Yat-Sen University, Guangzhou, China; ^2^ Department of Anesthesiology, State Key Laboratory of Oncology in South China, Sun Yat-Sen University Cancer Center, Guangzhou, China

**Keywords:** giant cell tumor, pulmonary metastasis, risk factor, nomogram, predictive model

## Abstract

**Purpose:**

Giant cell tumor of bone (GCTB) is an intermittent tumor with a low probability of pulmonary metastasis. Our aim was to investigate the risk factors and establish a nomogram predictive model for GCTB pulmonary metastasis.

**Methods:**

We retrospectively evaluated GCTB patients at our center from 1991 to 2014. The cohort was randomized into training and validation sets. Univariate and multivariate analyses were used to evaluate the risk factors of pulmonary metastasis. A nomogram was established. Internal validation was achieved based on ROC curve and C-index values in the validation set. Decision curve analysis was performed to assess the clinical performance of the nomogram.

**Results:**

417 patients were studied, including benign and malignant GCTBs. The average follow up was 79 months. Pulmonary metastases were observed in 27 cases. Four independent risk factors were identified: malignancy, tumor bearing time, times of recurrence and tumor size. A nomogram was developed to predict pulmonary metastasis with C-index values of 0.857 and 0.785 in the training and validation groups. In the decision curve analysis, patients could benefit from the nomogram, which differentiates patients at high risk for pulmonary metastasis and avoids unnecessary examination. According to the nomogram, patients with final risks of more than 0.06 should be scheduled for further chest scans.

**Conclusion:**

Malignancy, tumor bearing time, times of recurrence and tumor size were independent risk factors for pulmonary metastasis in GCTB patients. The nomogram can accurately predict the risk of pulmonary metastasis and help doctors to make clinical decisions for further chest examinations.

## INTRODUCTION

Giant cell tumor of bone (GCTB) is a relatively uncommon intermediate bone tumor with strong local aggressiveness. GCTB accounts for approximately 3%-5% of primary bone tumors and shows a relatively high incidence in Chinese populations [1, 2]. However, few GCTBs (1%-6%) transform into malignancies, particularly after radiotherapy or multiple local surgeries and recurrences [3–5]. Finch and Gleave first described pulmonary metastasis in benign GCTB, prior to which metastasis was only associated with malignant GCTBs [6]. Distal metastasis of benign GCTBs occurs in various sites, including the lung, liver and skin, and pulmonary metastases are most commonly observed [7–10]. Reflecting the low incidence of GCTBs and the pulmonary metastasis of this tumor, studies of the etiology or risk analysis of this disease are limited. Some authors have suggested that vascular invasion and iatrogenic embolization might be attributed to metastasis, although additional evidence is needed to confirm this hypothesis [11–14].

Outcomes of the pulmonary metastasis of GCTBs vary from spontaneous regression to uncontrolled growth with eventual death. The major treatment for pulmonary metastasis is surgical resection, although other options, including chemotherapy, radiation therapy and non-operative management, are also considered. The early diagnosis of pulmonary metastasis is vital to patient survival. Chest computed tomography (CT) is a common method to exclude pulmonary metastasis in patients with various malignancies; however, this technique is not regularly recommended to GCTB patients, as this tumor is not a malignant disease, which may lead to the delayed diagnosis and treatment of pulmonary metastasis. Additionally, chest CT scans may be administered late in the course of disease when GCTB patients already show typical manifestations. Several risk factors for pulmonary metastasis have been reported but the results of previous clinical studies have been inconsistent and could not provide guidance or practical clinical advice [15–19]. Thus, systemic risk evaluations and objective suggestions for lung screening in GCTB patients are needed.

A nomogram is a graphic tool for individual clinical outcome prediction based on a statistical formula. Nomograms have been widely used in oncology, as this technique has the advantage of visual simplicity and predictive accuracy [20]. In the present study, we explored the risk factors for the pulmonary metastasis of GCTBs and developed a nomogram predictive model based on these factors. Using this nomogram, we could distinguish GCTB patients with relatively high risks of pulmonary metastasis, which may contribute to the early diagnosis and treatment of these tumors.

## RESULTS

### Patients characteristics

A total of 509 patients were diagnosed with benign or malignant GCTB and received standard treatment at our center from 1991 to 2014. Seventy-five patients were either lost to follow up or followed for less than 2 years, and fifteen patients had combined severe pulmonary disease or other malignant tumors. A total of 417 patients with complete clinical data were included in the present study, among which 215 (51.6%) male and 202 (48.4%) female patients with an average age of 32 years old were included. The average time of follow up was 79 months (range, 24 to 312 months). Tumor locations were 74 (17.7%) in the axis and 343 (82.3%) in the extremity. The average tumor size was 6.2±2.3 cm, and the tumor bearing time was 9.1±12.3 months. A total of 12 patients (2.9%) were diagnosed with malignant GCTB by preoperative biopsy and postoperative specimens. All patients received wide or radical resection of the tumor and reconstructive surgeries where indicated. Among the 405 benign GCTB patients, 240 (59.3%) patients received intralesional aggressive curettage, while the remaining patients (40.7%) received marginal resection and reconstruction. A total of 7 patients accepted postoperative radiation therapy, and 165 patients experienced local recurrence 197 times either before or after treatment at our center.

Pulmonary metastasis was observed in 27 patients (6.5%) based on chest CT scan and diagnosed by a multidisciplinary team. Among these 27 patients, 4 patients had malignant GCTB in a primary location. The treatments for pulmonary metastatic foci were surgical resection for 19 cases, chemotherapy for 4 cases, RANKL inhibitor administration for 2 cases and observation for the remaining 2 cases. Pulmonary metastases were confirmed based on postoperative pathology specimens from surgical patients. During follow up, pulmonary lesions were under control in 19 patients, while 4 patients showed disease progression and received repeated surgical resections. A total of 4 patients with malignant GCTB developed pulmonary metastasis and passed away due to tumor progression. No patients with benign GCTB died from pulmonary metastasis.

### Univariate and multivariate analysis

The patients included in the present study were randomly divided into the training set (313 cases) and validation set (104 cases) at a ratio of 3:1. The clinical characteristics of the patients did not differ significantly between the two groups (p>0.05) (Table 1). The pulmonary metastasis rate was 6.1% in the training group and 7.7% in the validation group. Table 2 shows the relationships between clinical factors and pulmonary metastasis of GCTB in the univariate analysis. Age, sex, pathological fracture, tumor location and surgery type were found to be not relevant (p>0.05). Longer tumor bearing time was observed as relevant to pulmonary metastasis (p<0.001). Recurrence was also correlated, particularly when GCTB recurred more than once in the same location. A tumor size larger than 6 cm in maximum diameter, histological malignancy and Campanacci stage III were also observed as risk factors in univariate analysis (p<0.05). In multivariate analysis, malignancy, tumor bearing time, times of recurrence, and tumor size were independent risk factors of pulmonary metastasis in GCTB patients (Table 3).

**Table 1:** Variables in the training group and the validation group

Characteristics	Training	%	Validation	%	P value
**Age at diagnosis**	32.79±12.03		32.33±10.82		0.728
**Sex**					0.258
Male	156	49.8	59	56.7	
Female	157	50.2	45	43.3	
**Tumor bearing time (months)**	9.38±13.00		8.27±9.71		0.419
**Recurrence**					0.649
0	187	59.7	65	62.5	
1	103	32.9	34	32.7	
2	19	6.1	5	4.8	
3	4	1.3	0	0	
**Pathological fracture**					0.421
No	265	84.7	92	88.5	
Yes	48	15.3	12	11.5	
**Malignancy**					0.738
No	303	96.8	102	98.1	
Yes	10	3.2	2	1.9	
**Campanacci grade**					0.608
1	37	11.8	11	10.6	
2	130	41.5	49	47.1	
3	146	46.6	44	42.3	
**Tumor location**					0.872
Axial bone	55	17.6	19	18.3	
Appendicular bone	258	82.4	85	81.7	
**Tumor size**					0.982
<6 cm	193	61.7	64	61.5	
≥6 cm	120	38.3	40	38.5	
**Surgery**					0.413
Curettage	175	55.9	65	62.5	
Wide resection	138	44.1	39	37.5	
**Lung metastasis**	19	6.1	8	7.7	0.560

**Table 2:** Univariate analysis

Variables	SE	OR	95%CI lower	95%CI upper	*p* value
**Age at diagnosis**	0.208	0.988	0.948	1.030	0.568
**Sex (Male versus Female)**	0.421	0.888	0.350	2.248	0.802
**Tumor bearing time**	0.012	1.043	1.019	1.068	0.000
**Recurrence**					0.000
1 versus 0	1.255	2.200	0.719	6.729	0.167
2 versus 0	5.625	8.044	2.043	31.670	0.003
3 versus 0	32.661	30.167	3.614	251.828	0.002
**Pathological fracture**	0.304	0.292	0.038	2.240	0.236
**Malignancy**	5.661	7.688	1.816	32.550	0.006
**Campanacci grade (III versus I&II)**	1.171	2.609	1.082	6.289	0.033
**Tumor size (>6 cm versus ≤6 cm)**	2.657	4.966	1.740	14.170	0.003
**Tumor location**	0.459	0.787	0.251	2.470	0.681
**Surgery type (Wide resection versus Curettage)**	0.289	-0.071	-0.680	0.537	0.818

**Table 3:** Multivariate analysis

Variables	SE	OR	95%CI lower	95%CI upper	*p* value
**Malignancy**	0.911	2.052	0.265	3.839	0.024
**Tumor bearing time**	0.014	0.047	0.020	0.075	0.001
**Times of recurrences**	0.319	0.813	0.019	1.439	0.011
**Tumor size**	0.593	1.118	-0.044	2.280	0.049

### Development and validation of the predictive nomogram

**Figure 1:**
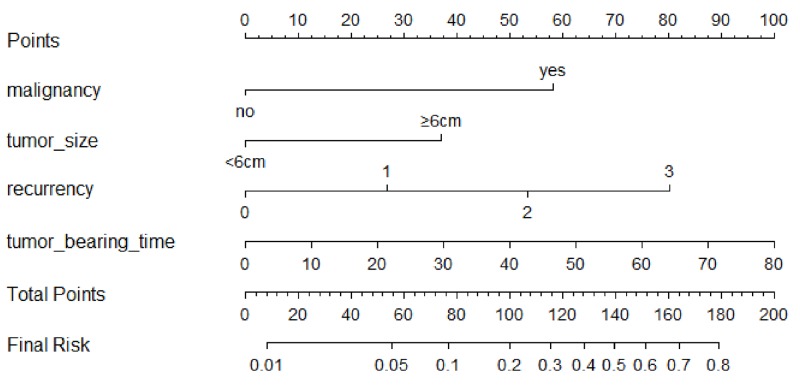
Nomogram for pulmonary metastasis in GCT patients

**Figure 2:**
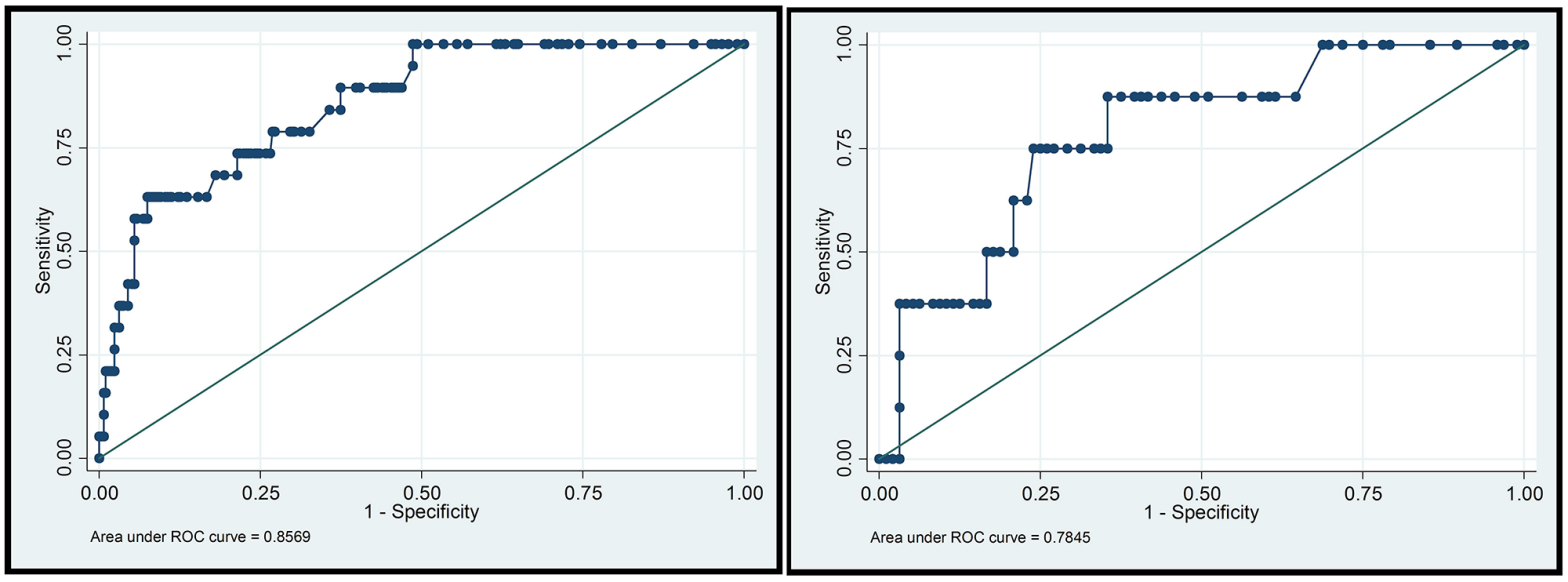
ROC curves in the training group and validation group In the training group, the C-index = 0.8569, 95%CI [0.779,0.935]. In the validation group, the C-index = 0.7845, 95%CI [0.626,0.943].

**Figure 3:**
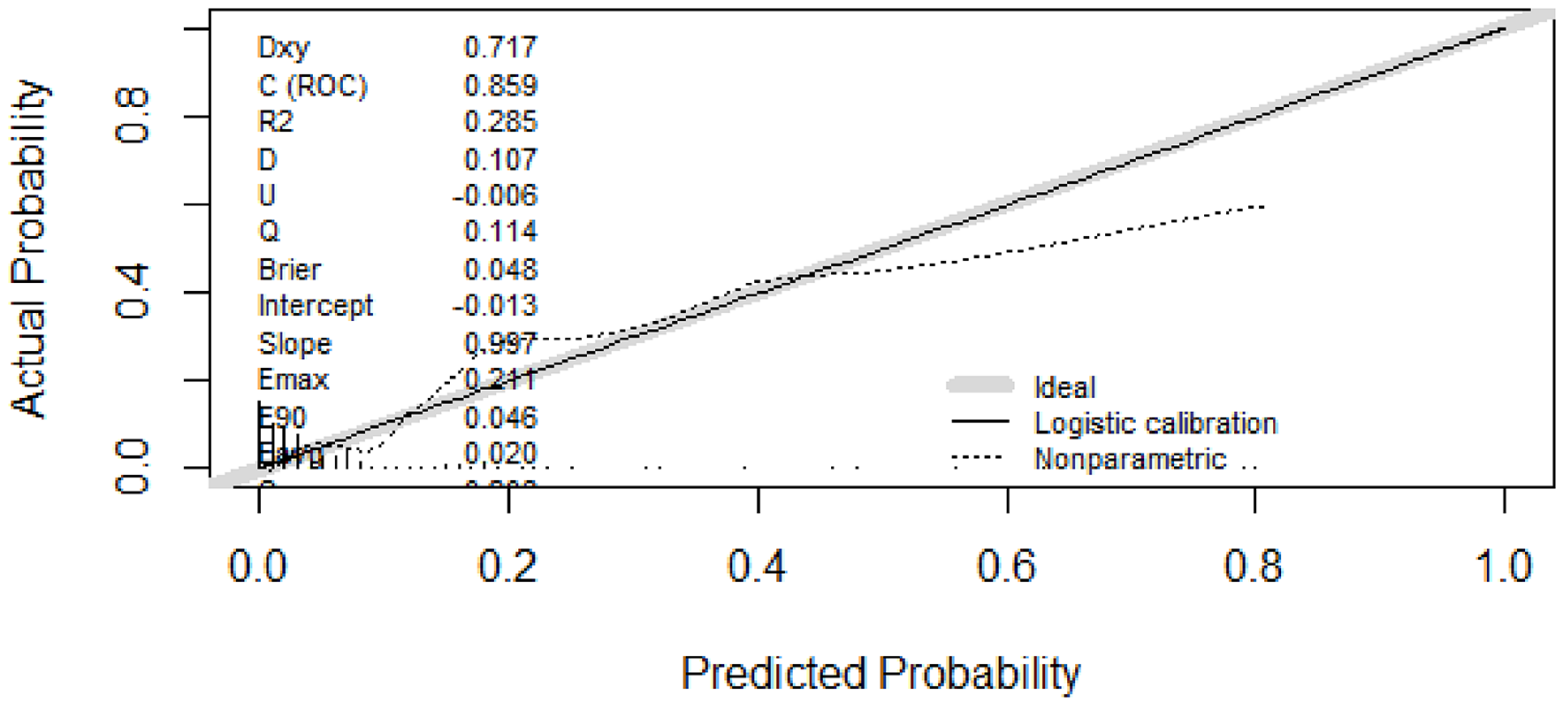
Calibration curve Consistency of the predictive and actual values was observed in the area of 0 and 0.5. As the majority of patients used to establish the model were of low risk (0 to 0.1), the model tended to show a relatively lower predictive value in extremely high-risk populations; this should not affect the clinical utility of the model because further examinations are strongly recommended for these patients.

A predictive model of pulmonary metastasis of GCTB was developed based on the above independent risk factors using logistic regression (Figure 1). Each level of single variable was assigned a specific score, and the total score for all risk factors indicated a specific predicted risk determined using the nomogram. According to the nomogram, malignancy in histology was primarily attributed to pulmonary metastasis as a single factor, while a tumor bearing time beyond 50 months and recurrence more than twice could be considered the same or even riskier. Tumor size had the least effect on pulmonary metastasis compared with that of other independent risk factors. The discrimination ability of the nomogram was evaluated based on ROC curves and C-index values. In the training and validation groups, the C-index values were 0.857 and 0.785, respectively, and the 95% confidence intervals were (0.779, 0.935) and (0.626, 0.943), respectively (Figure 2). Calibration in the validation group showed consistency between the predicted value and the ideal reference line (Figure 3).

**Figure 4:**
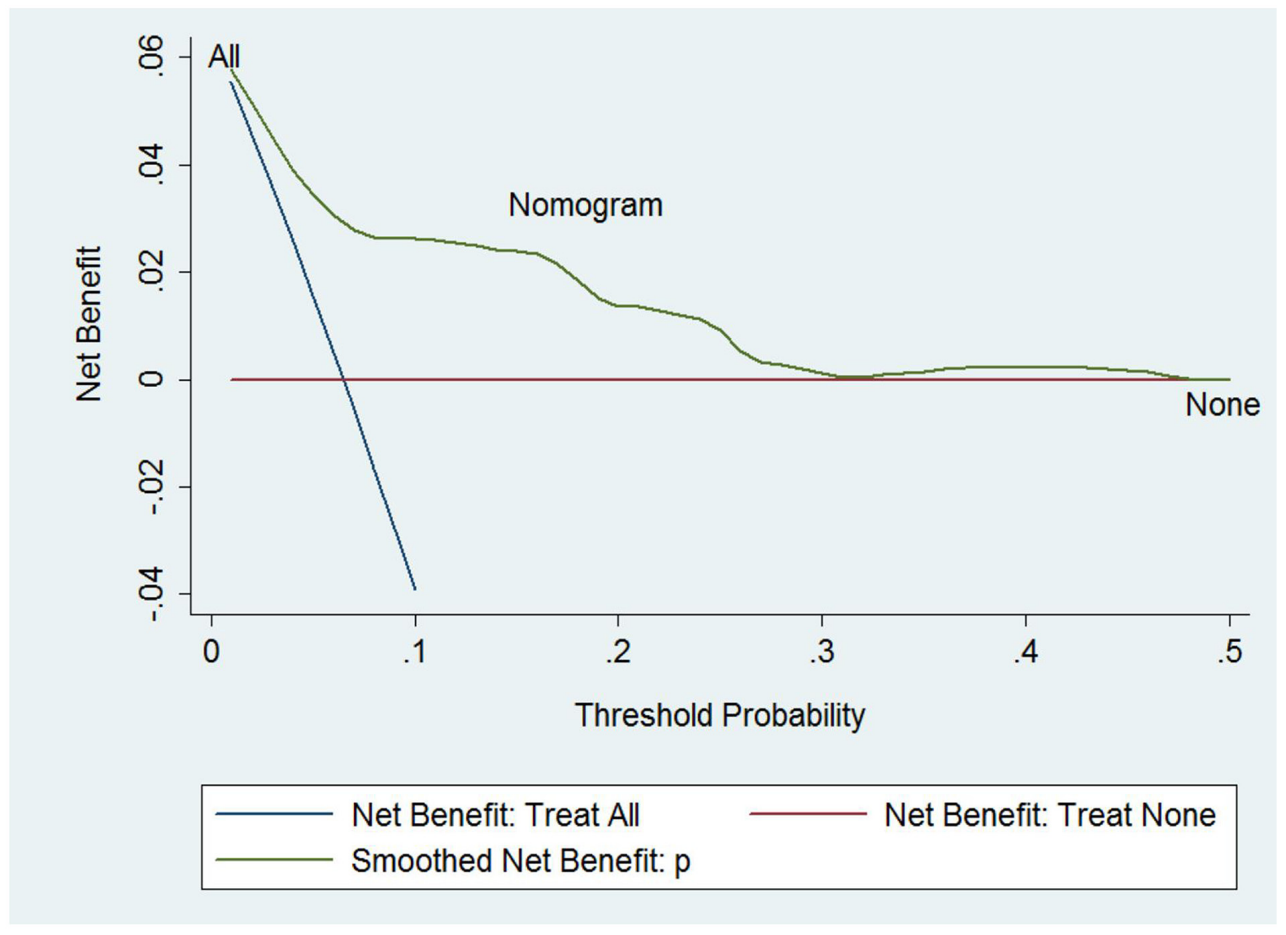
Decision curve analysis The treatment strategy was superior if it showed the highest value compared to the two simple strategies (i.e., performing screening for all patients (sloping solid line) or no patients (horizontal solid line)). For example, the value of net benefits would be 0.03 if we selected 10% as the cutoff value, suggesting that the nomogram would theoretically identify approximately 3 patients with GCTB lung metastasis among one hundred patients compared with simple observation, without adding any unnecessary tests (false positives).

**Figure 5:**
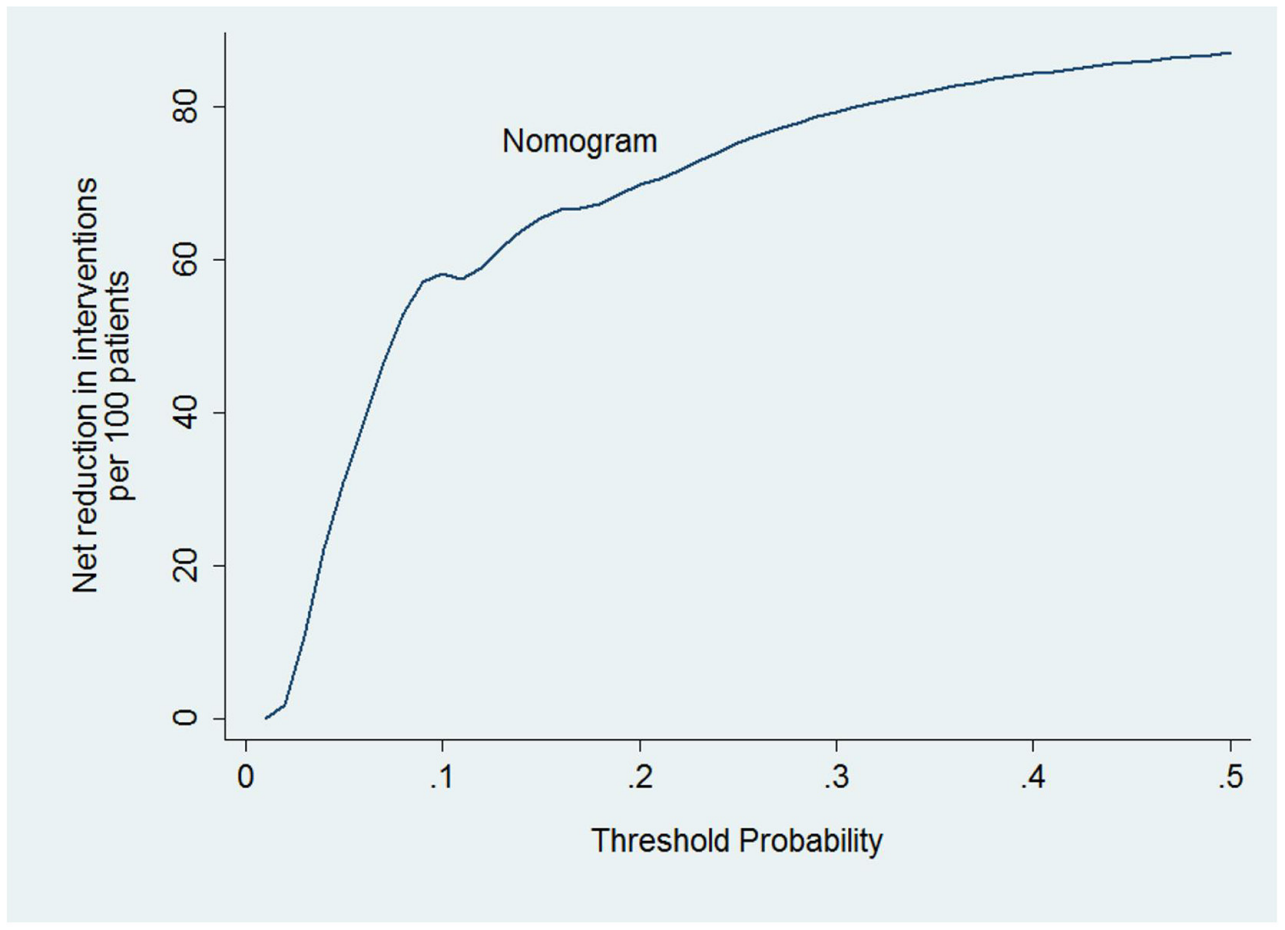
Net reduction When assessing the clinical utility of the nomogram, we also investigated whether this model would reduce unnecessary lung screening. At the same cutoff of 10%, the nomogram could reduce unnecessary test rates by 58%, without missing any metastasis.

To evaluate the clinical performance of the nomogram, we performed DCA, which focuses on the relative value of false-positive and false-negative values (Figure 4). The nomogram was compared using two simple strategies: all patients accepted chest CT scans or no patients accepted CT scans. Between the threshold probability of 0 and 0.3, which included most of the population, the nomogram was superior to these two simple strategies. The net reduction indicates the amount of unnecessary chest CT scans that the nomogram could reduce, without missing any metastasis when a specific threshold probability is shown (Figure 5). We propose that when the predicted value is higher than 0.06, chest CT scan should be recommended, as the net benefit and net reduction were both in a reasonable range. Under this cutoff value, the negative predictive value (NPV) was 97.1%, and the positive predictive value (PPV) was 17.4%.

## DISCUSSION

Although GCTB is classified as a benign lesion, this tumor has a certain potential for distal metastasis. Lung metastases had been reported to occur in 1% to 9% of all GCTB patients [21–23]. Pulmonary metastasis rate was 6.5% in our study, relatively higher compared to 2.1% in the large retrospective study containing 649 GCT cases [24].

Malignant GCTBs are extremely unusual, and most tumors develop secondary to radiotherapy or after repeated local recurrences. Few studies have addressed this event, considering that GCTBs should be treated as high-grade sarcomas due to similar biological behaviors, such as local aggressiveness and distal metastasis [3]. Twelve patients in the series examined in the present study were diagnosed with malignant GCTB after prudent multidisciplinary consultation. These patients were all treated and followed under the guidelines of osteosarcoma. Routine chest CT scan was recommended to all malignant GCTB patients, as the metastatic risk was much higher than in cases of benign GCTBs. In multivariate analysis, malignancy was also the most relevant independent factor of pulmonary metastasis. Patients with malignant GCTB were at six-fold increased risk to develop pulmonary metastasis compared with that of those with benign GCTBs. To emphasize the hazard of GCTB malignant transformation and provide a positive control to evaluate the effects of other risk factors, we assigned malignancy as the first risk factor in the nomogram in the present study.

Tumor recurrence has been identified as a risk factor of pulmonary metastasis in GCTBs [17, 22, 25–27]. We observed that the risk of pulmonary metastasis was positively correlated with the times of tumor recurrence. Benign GCTB patients who experienced more than 2 tumor recurrences showed a risk of pulmonary metastasis equal to or even higher than that of patients with malignant GCTBs. Authors who consider tumor recurrence as a risk factor of pulmonary metastasis have proposed that surgical manipulation can facilitate tumor immigration, thereby promoting lung metastases. This idea may partly explain why patients become more vulnerable to pulmonary metastasis after several operations and instances of tumor recurrence. Nevertheless, some patients in our center developed pulmonary metastasis prior to receiving invasive treatments, suggesting that there may be other potential mechanisms of pulmonary metastasis in GCTB.

In addition, tumor bearing time was also associated with pulmonary metastasis in the present study, independent of tumor recurrence. As reported in other centers, pulmonary metastases of benign GCTBs differ from those in malignant tumors, typically progressing slowly and seldom leading to severe symptoms until expanding to certain extents; some tumors might even shrink and fade after removal from the primary site [22]. Thus, we assumed that the longer a patient carries the primary tumor, the higher the chance for the tumor to gain access to vessels and hematogenously migrate to distal sites, such as the lungs. This novel finding and hypothesis was supported by clinical data; when the tumor bearing time was longer than four years, the chance of pulmonary metastasis was equal to that observed for malignant GCTBs.

Some authors have suggested that the Campanacci grade is associated with pulmonary metastasis [19], although we did not find this relation in the present study. However, tumor size was observed as an independent risk factor, reflecting tumor extent and local aggressiveness, and tumor size is much easier to measure and thus more objective. Age and tumor location did not manifest independent effects on pulmonary metastasis in the present study, while in other studies these factors were correlated. Although different treatments, including curettage, marginal resection and radiotherapy, have different impacts on local control, this parameter was not an independent risk factor of pulmonary metastasis in the present study.

Although pulmonary metastasis of GCTBs has been described and studied for years since the 1940s, the mechanism and risk factors have not yet been uncovered due to the low incidence of the disease itself and the low incidence of pulmonary metastasis. Clinical retrospective studies have attempted to answer these questions. Chan et al considered age, campanacci stage and recurrence were related to pulmonary metastasis, while none of these factors were associated in multivariate analysis [18]. Seethalakshmi Viswanathan el at found site of primary tumor, local treatment and local recurrence not related to pulmonary metastasis [15]. K.A. Siebenrock et al reported local recurrences and primary lesion at distal radius associating with pulmonary metastasis [28]. These results have been inconsistent and could not provide guidance or practical clinical advice. As an effective tool in risk prediction, nomograms have been applied in outcome prediction in various diseases. This technique maximizes the predictive accuracy and reflects the contribution of variables to the outcome visually and directly [20, 29]. In the present study, we successfully established a nomogram to predict pulmonary metastasis in GCTB based on the independent risk factors observed in multivariate analysis. The performance of this model in the training and validation groups was similar (C-index values of 0.857 and 0.785, respectively), and in both groups, the nomogram showed good predictive value.

After the evaluation of metastasis risk, a critical question is whether a patient needs further radiological examination of the lungs to exclude pulmonary metastasis. The nomogram showed good clinical performance in DCA, suggesting that decisions should be made according to the nomogram, as additional pulmonary metastases could be detected without extra unnecessary examinations. Furthermore, combining the Youden index, the DCA and clinical practice, 0.06 was identified as a proper cutoff value for further radiological examination, consistent with the incidence of pulmonary metastasis in the patient series examined in the present study. As the predictive values of pulmonary metastasis in most GCTBs were lower, the cut value of 0.06 highlights the fewer number of patients in the high-risk group, thus increasing the positive detection rate.

To our knowledge, this study shows the first nomogram prediction model for the pulmonary metastasis of GCTBs. This nomogram includes four clinical variables, obtained from preoperative examinations. The C-index values of the nomogram were 0.857 and 0.785 in training set and validation set, respectively. Thus, this model is reliable and useful, particularly for distinguishing benign GCTB patients with a relatively high risk of pulmonary metastasis, can help the surgeon with early diagnosis and treatment of metastatic foci, which may be ignored in clinical practice, and will hopefully improve the outcome of both benign and malignant GCTBs.

Despite the good performance of the nomogram, its major limitation was that the clinical data included in this research were obtained from a single center in China. Thus, external validation is required prior to the application of this method in other centers or populations.

In conclusion, malignancy, times of recurrence, tumor bearing time and tumor size were identified as independent risk factors of pulmonary metastasis in GCTB patients. The nomogram based on these risk factors could objectively and accurately predict pulmonary metastasis in GCTB patients. Clinical application of this nomogram may aid the surgeon to distinguish patients at high risk of pulmonary metastasis and avoid unnecessary further examination.

## MATERIALS AND METHODS

### Patients

Patients diagnosed with GCTBs and receiving treatments at the Musculoskeletal Tumor Center of the First Affiliated Hospital of Sun Yat-sen University from 1991 to 2014 were retrospectively reviewed. The following criteria for case inclusion were considered: (1) GCTBs histologically confirmed using core needle biopsy; (2) recurrent GCTBs with previous operation history; (3) GCTBs located in either axis or extremity; and (4) malignant GCTBs with or without a previous history of benign GCTB and radiotherapy. The following exclusion criteria were considered: (1) patients with previous lung diseases; (2) history of any other malignancies; and (3) patients who had been followed for less than two years. Subsequently, 75% of the included patients were randomly selected as the training set to explore risk factors and develop the predictive model. The remaining 25% of the included patients were selected as the validation set to validate the nomogram model and assess its predictive power. The Institutional Ethical Board approved the present study.

### Diagnosis and treatment

Diagnoses of GCTB and pulmonary metastasis were based on multidisciplinary teamwork. Radiology analyses, including X-ray, CT and (or) magnetic resonance imaging (MRI), were performed for local lesions to evaluate aggressiveness. Preoperative core needle biopsy of the lesion was performed to distinguish benign and malignant tumors, and biopsy was also performed to confirm disease recurrence and malignant changes. Multi-slice spiral CT was employed to evaluate pulmonary metastasis for malignant or benign GCTB with suspicious findings in clinical practice. A maximum diameter of pulmonary lesions > 1 cm and/or progression of the lesion based on serial CT images were considered to indicate pulmonary metastasis, with the exclusion of potential diagnoses other than metastasis [19]. The local treatment of benign GCTB consisted of intralesional aggressive curettage with adjuvant therapy, including liquid nitrogen, cryoablation and a high-speed drill; marginal resection was occasionally performed according to the extension of the tumor. Wide resection or even radical resection was performed in malignant GCTBs.

### Follow up

Patients were scheduled for follow up. Local X-ray and MRI scans were employed to exclude recurrence, which was confirmed by core needle biopsy. A chest CT scan is routinely recommended for malignant GCTB patients, while benign GCTB patients receive chest CT scans only if there is evidence of pulmonary metastasis. The time of follow up was defined as the time from confirmed diagnosis to the latest follow up.

The data reviewed included clinical characteristics, radiology features and histology diagnosis. Tumor locations were separated into axis (vertebrae, pelvis or scapulae) and extremity. Campanacci staging [1] and tumor size were used to describe the extension of local lesions. Malignancy referred to tumors those were diagnosed as malignant GCTB at first biopsy of the tumor or those developed malignant transformation during disease progression. Criteria of malignant GCTB diagnosis was as described by Bertoni F [3]. The tumor bearing time was defined as the interval between tumor emergence and local clearance of the tumor, including the interval between tumor recurrence and re-control of the local lesion.

### Statistical analysis

Continuous variables were expressed as the means±SD, and categorical variables were expressed as numbers. The student's t-test and chi-square test (or Fisher's exact test) were used to compare variables between groups. Factors significant in univariate analysis were included in multivariate logistic regression to identify independent variables. The establishment of the predictive model was based on the independent factors from multivariate analysis. The discrimination power of the model was evaluated based on the receiver operating characteristic (ROC) curve and concordance index (C-index) values, which were identical to the nonparametric area under the ROC curve. C-index values ranged from 0 to 1, with 1 indicating perfect prediction and 0.5 indicating random prediction. Calibration was performed for internal validation of the predictive model, which represents the fitness of predictive and actual values. Finally, a decision curve analysis (DCA), as described by Vickers et al., was used to assess the clinical utility of the model by calculating the net benefit considering different threshold probabilities [30]. SPSS (version 19.0, Chicago, IL, USA) for Windows and rms package in R version 3.2.0 were used for statistical analysis. A p value less than 0.05 was considered statistically significant.

## Abbreviations

GCTB: Giant cell tumor of bone
CT: Computed tomography
MRI: Magnetic resonance imaging
ROC: Receiver operating characteristic
C-index: Concordance index
DCA: Decision curve analysis
NPV: Negative predictive value
PPV: Positive predictive value
